# Comment on “Can Artificial Intelligence Be Successful as an Anaesthesiology and Reanimation Resident?”

**DOI:** 10.4274/TJAR.2025.252040

**Published:** 2025-05-30

**Authors:** Hinpetch Daungsupawong, Viroj Wiwanitkit

**Affiliations:** 1 Private Academic Consultant, Phônhông, Lao People’s Democratic Republic; 2 Department of Research Analytics, Saveetha Dental College and Hospitals, Saveetha Institute of Medical and Technical Sciences, Saveetha University, Chennai, India

**Keywords:** AI, anaesthesiology, intensive care, resident, technology

## Dear Editor,

The publication on “Can Artificial Intelligence Be Successful as an Anaesthesiology and Reanimation Resident?”^1^ The purpose of this study was to compare ChatGPT artificial intelligence’s (AI) performance with that of anaesthesiology and critical care residents using an exam equivalent to the European Diploma in Anaesthesiology and Intensive Care Part 1. Although the study’s design indicates an attempt to examine the possibilities of AI in a complex medical setting, there are various flaws in the research methodology that should be carefully considered. For example, asking ChatGPT the identical questions a day in advance may increase the risk of data leakage, or the model may alter the responses based on context. When contrasted to people who do not have time to prepare in advance, those who do have time exhibit a distinct disparity.

In terms of statistics, non-parametric data should be reported using the median and range. However, the study did not identify the number of test takers, which influenced the statistical analysis. For example, the *P* value, indicating a significant difference between the training groups of less than and more than 24 months, limited the ability to thoroughly investigate the effect size. Furthermore, within the human group, the ranking of ChatGPT lacked a defined criterion, making it difficult to examine its location in relation to the score distribution in each group.

To encourage in-depth conversation. Questions to consider include: How does ChatGPT employ data sources and processing methods to provide answers? Should there be special criteria or models for assessing AI medical expertise that differ from human knowledge?

Does the fact that ChatGPT performed modestly among trainees imply that AI can replace basic analytical tasks? What are the implications for patient safety if AI is using old or out-of-date data? These questions will spark ethical debates and future regulation of AI therapeutic applications.

Future research should focus on evaluating AI in clinical scenario simulations that demand contextual analysis or time-bound decision-making, rather than answering multiple-choice questions that do not reflect practical skills. Furthermore, AI should be compared to healthcare professionals at various levels of expertise (e.g., senior physicians or specialists) and evaluated in terms of communication, data interpretation, and treatment recommendation capabilities to determine AI’s true potential and limitations in supporting responsible medical practice.
